# Toxic Metal Concentrations in Drinking Water and Possible Effect on Sex Hormones among Men in Sabongida-Ora, Edo State, Nigeria

**DOI:** 10.3390/medicines9010004

**Published:** 2022-01-07

**Authors:** Osaro Ogie Enehizena, Mathias A. Emokpae

**Affiliations:** Department of Medical Laboratory Science, School of Basic Medical Sciences, University of Benin, Benin 300283, Nigeria; enehizenaosaro@gmail.com

**Keywords:** male, drinking water, gonadal steroid hormones, Nigeria

## Abstract

Drinking water can be a potential source of toxic metals, which are a known leading cause of infertility in men. This study determines the concentrations of lead (Pb), cadmium (Cd), zinc (Zn), copper (Cu) in drinking water (borehole, hand-dug well and treated water) and sex hormone levels (serum follicle stimulating hormone (FSH), luteinizing hormone (LH), prolactin (PROL), estradiol (E2), progesterone (PROG), and testosterone (T) in males who drink water mainly from these sources. The concentrations of Cd, and Pb in hand-dug wells were higher than the permissible limit recommended by the World Health Organization (WHO) while Zn and Cu were within the permissible levels in drinking water. Blood Cd and Pb levels were significantly higher (*p* < 0.001) among subjects who consumed hand-dug and borehole water than treated water, while serum Zn was significantly lower (*p* < 0.001) in hand-dug well and borehole water consumers than in control subjects. Also, serum FSH (*p* < 0.001), LH (*p* < 0.001), E2 (*p* < 0.002), PROG (*p* < 0.04) and T (*p* < 0.001) were significantly lower among hand-dug well and borehole water consumers than controls, while PROL (*p* < 0.001) was significantly higher in hand-dug well and borehole water consumers than controls. Blood Cd and Pb levels were significantly higher (*p* < 0.001) in hand-dug well water consumers than borehole water consumers. The consumption of water from hand-dug wells may have adverse reproductive sequelae among consumers.

## 1. Introduction

Water safety and quality are very important for human development and well-being. Besides the pathogenic risk of harmful microorganisms, several chemical contaminants are present in water as a result of human activities (anthropogenic) which could be risk factors to human and animal health [1,2]. Some authors have indicated that the contaminants in water could impair development, fertility and reproductive function in non-human mammals, humans, and aquatic wild life. Others have observed that exposure to water disinfection byproducts in drinking water can lead to cardiac disorders in animal models [3,4]. Furthermore, exposure to bisphenol A (BPA) and phthalates are reported to cause decline in fecundity by premature activation of primordial follicles in mammals as well as changes in sex-steroid hormone levels [5,6,7,8,9]. Some have suggested that pesticides contaminants in drinking water might impact reproductive health. Studies have suggested that exposure to some pesticides was associated with low sperm quality and quantity as well as adverse pregnancy outcomes in both animals and humans [10,11].

The toxic metals found in drinking-water come from contamination of surface and ground waters by industrial sewage and agricultural runoff. In some communities where treated water distribution network is a challenge, those who cannot afford bottled or mineral water with controlled toxic metal concentrations have no alternative than to consume hand-dug well, borehole or tap water. Therefore, the possible contamination of drinking water with toxic metals and subsequent accumulation are greatly increased [12]. The quality control of drinking-water and detection of its toxic metals is an extremely critical issue in order to maintain sound human health [13]. Toxic metals can bioaccumulate in organisms because of their long half-life [14]. Toxic metals often bind to vital cellular components, such as structural proteins, enzymes, and nucleic acids, and interfere with their functions [15]. Symptoms and effects can vary according to the metal or metal compound and the dose involved. Generally, long-term exposure to toxic metals can have carcinogenic, central and peripheral nervous system and circulatory effects.

Circulating levels of the follicle stimulating hormone (FSH), the luteinizing hormone (LH), prolactin and testosterone are vital for spermatogenesis and sexual function. The accumulation of toxic metals in the body is harmful to sexual function and reproduction. Therefore, the evaluation of toxic levels in drinking water and the possible effect on reproductive hormones is important for public health information. 

In developing countries like Nigeria, the quality of drinking water is increasingly contaminated and hazardous to human health because of high population growth, expansion in industries, indiscriminate dumping of waste and chemical effluents in to canals and other water sources [16]. Some authors have suggested that frequent studies of the quality of drinking water should be undertaken. Since groundwater is directly in contact with soil, rocks, and plants, the constituents of these sources might contaminate the groundwater [17,18,19]. The quality control of drinking-water and the assessment of its toxic metals contents is extremely critical and is of public health importance. Studies have indicated that male infertility due to endocrine abnormalities is common in Nigeria [20,21,22] and the exact causes are not known. This study seeks to determine the concentrations of Pd, Cd, Zn and Cu in hand-dug well, borehole and treated water, the blood levels of these metals as well as sex hormones in subjects who drink water solely from these sources. This will provide evidence to indicate if the levels of these metals in the drinking water are higher or within the WHO permissible levels and their possible association with sex hormones among the subjects who drink water solely from these sources.

## 2. Materials and Methods

### 2.1. Study Area and Water Sample Collection

The study was conducted at Sabongida Ora, Owan West Local Government Area of Edo State among men who drink water solely from hand-dug well, borehole and treated sources. 

Water samples were randomly selected across ten (10) locations in Sabongida-Ora, 15% were from Ohia, 10% from Borehole road, 5% were from Holy Trinity Grammar School Road, 20% from Uhonmora, 20% from Evbiobe,10% Living Faith Church, Sabongida-Ora Road, 5% were from Oke, 5% were from Ovbiokhuarin and 5% were from Eme and 5% from Owan West Local Government Secretarial. Water samples were collected in clean-glass containers washed by soaking in 20% (*v*/*v*) Nitric acid for 24 h and rinsed with several changes of tripled distilled water and dried in a polypropylene container. Sampling was conducted early in the morning before water abstraction commenced by residents. Two separate samples per water source were collected for analysis.

### 2.2. Sample Size Determination

**Figure 1:**
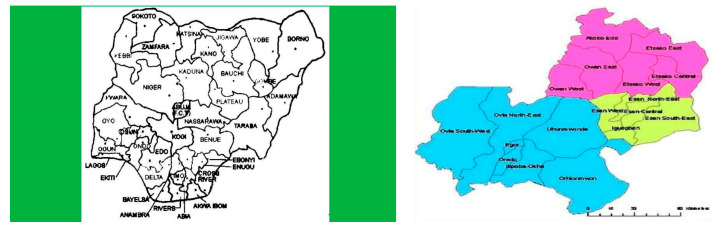
Map of Nigeria and Edo State in Nigeria.

The sample size for hormonal evaluation was determined using the sample size determination formula for Health studies N = Z^2^P(1 − P)/d^2^ [23] where n = minimum sample size, P = estimated prevalence, Z = standard normal deviate that corresponds to 95% confidence limit (1.96), d^2^ is the alpha level of significance (5%) and the prevalence of hormonal abnormalities in occupationally exposure subjects to toxic metal contamination (96.7%) [24]. As a result, a minimum of 60 participants and 30 non-occupationally exposure healthy subjects were enrolled in the study. Figure 1 shows the location in Nigeria where the study was conducted.

Ten boreholes and ten hand dug well water from different locations in the town were evaluated for metal element levels. Sixty individuals (study group) who have been drinking water from these sources for a minimum of one year and thirty individuals of the control group were evaluated for cadmium, lead, zinc, copper and sex hormone levels.

### 2.3. Research Design

This is a prospective case-control study of male participants who consumed drinking water from hand dug wells, boreholes, and also drank treated water. Blood samples were collected from participants in the morning.

### 2.4. Inclusion and Exclusion Criteria

Healthy men within the reproductive age of 20–45 years and drank water solely from hand dug wells and consumed borehole water and treated water were included in the study. Individuals on male contraceptives, or with testicular varicocele, had been on long-term medications, living with HIV, or had chronic and serious systemic illness, took steroid preparations, did not consent and were smokers were excluded from the study.

### 2.5. Ethical Consideration

The protocol for the study was reviewed and approved by the Ethics Committee of the Edo State Ministry of Health (ethical code HM1208266, dated 31 July 2017). Informed consent was given by the participants before commencement of study.

### 2.6. Data Collection Tools and Techniques

The socio-demographic data were collected using a semi-structured questionnaire. The questionnaire was distributed among male participants drinking water from hand dug well and borehole water as well as those who consume treated water.

### 2.7. Laboratory Analysis

#### Estimation of Cadmium, Lead, Zinc and Copper

##### Procedure

Exactly 1 mL of whole blood sample was measured and poured into the khedjahl digestion tube, 5 mL of the mixed acid (Nitric-Perchloric acid mixture. Ratio 2:1) was added. Digestion tube was brought to the heater and heated. When dense white fume occurred, heating continues until a clean solution was obtained. It was removed from the heater, cooled, and a small amount of deionized water was added. The solution was filtered with Whiteman no 42 filter paper into a 25 mL volumetric flask, and the volume was made up with deionized water to 25 mL. A reagent blank was prepared identically but without the blood sample. The metal concentrations were determined by an Atomic Absorption Spectrophotometer (BUCK Scientific, Norwalk, CT, USA) according to the manufacturer’s protocol. 

### 2.8. Standard Preparation (QC)

The AAS was first calibrated using Buck certified atomic absorption standards for the respective metals to obtain a calibration curve. The reagent blank was run at intervals of every 10 samples of analysis to eliminate equipment drift. All samples were analyzed in duplicate for reproducibility, precision and to ensure accurate checks.

### 2.9. Determination of Sex Hormones

The sex hormones (luteinizing hormone, follicle stimulating hormone, prolactin, testosterone, estradiol and progesterone) were assayed by ELISA technique (Monobind Inc. Lake Forest, CA, USA (Accu-Bind ELISA Microwells)).

### 2.10. Data Analysis

Data analysis was done using the statistical software SPSS version 21 (SPSS Inc., Chicago, IL, USA). The Student’s *t*-test and Chi-square test were used to compare variables where appropriate, and a *p* < 0.05 was considered statistically significant.

## 3. Results

The age distribution of the 90 male subjects in this study shows that a majority (62.2%) were between the ages of 18–25 years, while the least (6.6%) were 40 years and above; 3.3% were underweight (BMI of less than 16.5 kg/m^2^), 35.5% were overweight (BMI of 28.6 kg/m^2^ and 14.4% were obese (BMI of 33.5 kg/m^2^) (Table 1).

Table 2 indicates that the concentrations of Cd and Pb in hand-dug well and borehole water were higher than the World Health Organization (WHO) permissible limits in drinking water while the concentrations of Zn and Cu were within the WHO permissible limits [18].

The levels of Cd and Pb were significantly higher (*p* < 0.001) in hand-dug well and borehole water consumers, while Zn was significantly lower (*p* < 0.001) in hand dug well/borehole water consumers than treated water consumers (Table 3).

The comparison of blood Cd, Pb, Cu and Zn among consumers of hand-dug well water and borehole water shows that Cd and Pb were significantly higher (*p* < 0.001) among hand-dug well water consumers than borehole water consumers while Cu and Zn were significantly lower in hand-dug well water consumers than borehole water consumers (Table 4).

Table 5 shows that serum FSH (*p* < 0.001), LH (*p* < 0.001), estradiol (*p* < 0.002), progesterone (*p* < 0.04), and testosterone (*p* < 0.001) were significantly lower among hand-dug well/borehole water consumers than treated water consumers. Conversely, serum prolactin was significantly higher (*p* < 0.001) among hand-dug well/borehole water consumers than treated water consumers. Serum FSH, LH, estradiol, testosterone and prolactin were significantly lower (*p* < 0.001) among hand-dug water consumers than borehole water consumers (Table 6).

Table 7 shows the interrelationship of FSH and LH with toxic and essential metals, age and BMI of participants. Serum FSH correlated negatively (r= −0.398; *p* < 0.001) with Pb, and positively with serum Zn (r = 0.422; *p* < 0.002). Similarly, LH correlated negatively with Cd (r = −0.622; *p* < 0.001) and positively with Zn (r = 0.745; *p* < 0.001). No significant correlation was observed between FSH, LH and the other measured variables. The regression coefficient between serum estradiol and other measured parameters indicates that estradiol correlated positively with Zn (r = 0.412; *p* < 0.003). There was no significant correlation between serum progesterone and other measured parameters. Similarly, serum prolactin correlated positively with Pb (r = 0.443; *p* < 0.006) and negatively with Zn (r= −404; *p* < 0.047). Lastly, serum testosterone correlated positively with Zn (r = 0.542; *p* < 0.001) (Table 8 and Table 9).

## 4. Discussion

Toxic metal contamination in drinking water is a potential health risk to humans and has been reported to be one of the root causes of many chronic health challenges, including cancer, infertility, and organ damage [25]. The presence of toxic metals and trace elements cannot be visualized with the naked eye, but are detected in water through laboratory tests. This study was conducted to determine Cd, Pb, Zn and Cu levels in hand-dug well, borehole and treated drinking water and the possible reproductive health effects on the consumers. In order to protect human health, guidelines for the presence of toxic metals and trace elements in drinking water have been given by international organizations such as the WHO and the European Union Commission [26]. These organizations prescribed that these chemical element levels in water should not be higher than the maximum permissible level in the water as specified. Maximum contaminant level is an enforceable standard in numerical range value to ensure no adverse effects on human health. The upper limit of the highest level of a contaminant is the maximum allowed in a water system for a particular chemical element.

The concentrations of Cd and Pb in hand-dug well and borehole water were higher than WHO-recommended permissible limits in drinking water [27]. This is consistent with previous studies in some parts of Nigeria. For example, Momodu and Anyakora [28] observed that 84.21% of underground water samples contained Cd concentrations higher than the maximum contaminant level (0.003 mg/L), with maximum concentrations of 0.098 mg/L (9.8 µg/dL). The reported concentration is higher than observed in this study (0.56 µg/dL). The detected level, which is higher than the maximum contaminant level, is of great concern, since Cd has the potential to cause male infertility [29], cancers [30], because of its long biological half-life [31], and it can bioaccumulate, leading to chronic organ damage [32]. 

The concentration of Pb detected in hand-dug well and borehole water in this study was also higher than the recommended permissible limit by WHO. The finding is consistent with that of Momodu and Anyakara [28] in Surulere, Lagos. The authors observed that 10 of the well water samples and 19 borehole water samples contained Pb, and six of the well water samples and 12 of the borehole water samples contained levels above the maximum contaminant level (0.01 mg/L), with the maximum concentration detected being 0.024 mg/L (2.4 µg/dL). These findings are of concern, since Pb can bioaccumulate, and it affects the body’s general metabolism [33], as well as male infertility [34]. Lead is also a neurotoxin and may be responsible for the most common type of human mental toxicosis [35]. Also, some authors have associated Pb exposures, even at low levels, with elevated blood pressure [36] and reduced intelligence quotients in children [37]. Lead is a naturally occurring metal present in small quantities in the Earth’s crust. Although lead occurs naturally in the environment, human activities such as the use of fossil fuel, mining and manufacturing industries contribute to the release of high concentrations of it. The concentration of Pb observed in hand-dug well and borehole water was higher than in treated water and more than the quantity recommended in drinking water by the WHO. The permissible limit recommended by WHO for drinking water is 0.01 mg/L. It has been emphasized that chemical contaminants in drinking water is a public health risk and may have immediate health effects [38]. These contaminants commonly occur due to natural and anthropogenic activities [39]. Natural sources of contaminants include host rocks which may be due to the geology of the area, volcanic activities, and chemical evolution, while anthropogenic activities commonly emanate from indiscriminate disposal of waste, urbanization, industrialization, mining and domestic activities, among others [40,41]. Some of the described mechanisms by which toxic metals impair reproductive health are ion mimicry, disruption of cell signaling pathways, epigenetic control of gene expression, oxidative stress, apoptosis, altered gene expression, inflammation, damaging of the testis-blood barrier and endocrine disruption. The relatively high levels of toxic metals in hand-dug wells and borehole drinking water may pose some reproductive health challenges. Apart from the observed abnormal levels of sex hormones in the participants, some authors have reported significantly higher levels of Cd and Pb in seminal plasma as well as poor semen quality among infertile men [29,34]. Female reproductive health is also affected by toxic metal accumulation. Cadmium has been reported to accumulate in human endometrial tissue, and can stimulate estrogen production by binding with alpha and beta estrogen receptors, as well as progesterone receptors, leading to estrogen dependent diseases (breast and endometrial cancers, endometriosis and spontaneous abortions). Similarly, toxic metal accumulation in children have been associated with mental retardation, neurocognitive disorders, behavioural disorders, respiratory diseases, cancer and cardiovascular diseases [6,19,21,42].

The significantly lower levels of Zn and Cu in those who drink hand-dug well and borehole water than control subjects may be due to the interaction of between Cd and essential elements. This interaction occurs at different stages of absorption, distribution in the body, and excretion of the elements at the stage of biological functions. Therefore, the zinc status of the body is especially vital in relation to accumulation of Cd in the body.

In this study, it was observed that the concentrations of Pb and Cd in the blood of hand dug well and borehole water consumers were higher than those consuming treated water in the same environment (controls). This finding is consistent with the other studies in Baghdad, Beirut, and Al-Najaf, which reported that the blood Pb and other toxic metals were higher among exposed individuals [43,44]. Copper and zinc were lower in the subject than the controls. The higher blood level of Cd and Pb among the subjects may be traceable to the drinking of contaminated water (hand-dug well and borehole water) since the respondents were non-occupationally exposed. The respondents reported that they have been consuming water from these sources for several years, it being the only source of drinking water during the study.

Significantly lower levels of sex hormones among the subjects that consume hand-dug well and borehole water than controls were also observed. From previous studies, it was observed that toxic metal contaminations are potent endocrine disruptors and oxidative stress inducers [45] as well as hypothalamo-pituitary-gonadal axis inhibitors [46,47,48]. These glands have been shown to act with a common function in order to achieve a common goal of regulating the reproductive and sex hormone production. The HPG axis plays a critical part in the development and regulation of a number of the body’s systems, such as the reproductive and immune systems. Any fluctuations in this axis causes changes in the hormones produced by each gland and has various local and systemic effects on the body. This axis controls development, reproduction and ageing in animals [49]. Gonadotropin-releasing hormone (GnRH) is secreted from the hypothalamus by GnRH-expressing neurons. The anterior portion of the pituitary gland produces luteinizing hormone (LH) and follicle stimulating hormone (FSH) and the gonads produce estrogen and testosterone. The male reproductive system depends upon the activities of these different hormones produced by various body glands for adequate physiological functions [50]. Testosterone helps to produce and maintain the secondary sexual characteristics of the male and is also responsible for the sex drive as well as work with the FSH to stimulate the production of sperm. If the sperm levels are high, the testes release inhibin which travels through the blood stream to the brain, where it prevents the secretion of GnRH. In the absence of GnRH, FSH, and LH levels fall and sperm production slows. This is one of the major feedback mechanisms which help to regulate and maintained sex hormones at relatively constant concentrations [51].

The data indicate that lead (Pb) was negatively associated with FSH and Estradiol and cadmium was negatively associated with LH. This result is in line with the study carried out by Telisman et al. [52]. Conversely, zinc correlated positively with FSH, LH, Estradiol and testosterone and negatively correlated (*p* < 0.05). Therefore, the observed decline in the serum levels of the reproductive hormones with increased levels of toxic metals suggests that toxic metals may have been responsible for the significantly lower levels of the hormones.

## 5. Conclusions

From this study, it is evident that consumption of contaminated water with toxic metals might pose a reproductive health threat to consumers. This alteration in reproductive hormone levels may cause subfertility and other disease conditions in men. In view of the dangers associated with the consumption of water contaminated with toxic metals and other impurities, it becomes necessary to ensure that water from hand-dug wells and boreholes should be treated before consumption. Such methods of water purification include physical methods (filtration, sedimentation or boiling and distillation), biological methods (sand filters, active carbon) and chemical methods (flocculation, chlorination and the use of ultraviolet light).

## Figures and Tables

**Table 1:** Age and body mass index of hand-dug well/borehole water and treated water consumers.

Variables	Total (n = 90)	Dug-Well and Borehole Water Consumers (n = 60)	Control Group (n = 30)	X^2^	*p*-Value
		Age (Years)			
18–25	56 (62.2%)	31 (51.6%)	25 (83.3%)		
26–35	20 (22.2%)	15 (25.0%)	5 (16.6%)		
36–40	8 (8.8%)	8 (13.3%)	0 (0.0%)	71.60	*p* = 0.001
40–above	6 (6.6%)	6 (10.0%)	0 (0.0%)		
		Body mass	Index (kg/m^2^)		
Underweight	3 (3.3%)	3 (5.0%)	0 (0.0%)		
Normal	42 (46.6%)	22 (36.6%)	20 (66.6%)		
Overweight	32 (35.5%)	23 (38.6%)	9 (30.0%)	45.53	*p* = 0.001
Obese	13 (14.4%)	12 (20.0%)	1 (3.3%)		

Chi-Square (X^2^) was used to compare the categorical variables.

**Table 2:** Toxic and essential metal concentrations in hand-dug well, borehole and treated water.

Sources of Water	Toxic and	Essential	Metals	
	Cd (µg/dL)	Pb (µg/dL)	Zn (µg/dL)	Cu (µg/dL)
Hand-dug well (n = 10)	0.51 ± 0.02	1.81 ± 0.10	215 ± 9.8	125 ± 10.2
Borehole (n = 10)	0.32 ± 0.01	1.10 ± 0.02	198 ± 10.2	105 ± 8.2
Treated water (n = 10)	0.29 ± 0.01	0.81 ± 0.10	192 ± 9.8	104 ± 7.9
WHO permissible limit in drinking water	0.3 (0.003 mg/L)	1.0 (0.010 mg/L)	300 (3.0 mg/L)	200 (2.0 mg/L)

Values are expressed in mean ± SD, Cd-cadmium, Pb-lead, Cu-Copper, Zn-Zinc.

**Table 3:** Levels of Blood Cd, Pb, Cu and Zn among Hand-Dug Well/Borehole Water Consumers and treated water consumers.

Measured Parameters	Hand-Dug Well/Borehole Water Consumers N = 60	Treated Water Consumers N = 30	*p*-Value
Cd (µg/dL)	3.62 ± 0.41	0.91 ± 0.21	*p* = 0.001
Pb (µg/dL)	3.89 ± 3.25	1.64 ± 0.04	*p* = 0.001
Cu (µg/dL)	97.03 ± 1.62	97.90 ± 2.63	*p* = 0.335
Zn (µg/dL)	98.26 ± 2.58	163.30 ± 3.43	*p* = 0.001

Value are expressed in mean ± SD, Cd-cadmium, Pb-lead, Cu-Copper, Zn-Zinc. *p* < 0.05—Significant.

**Table 4:** Comparison of Blood Cd, Pb, serum Cu and Zn levels in Hand-dug Well and Borehole Water Consumers.

Measured Parameters	Hand-Dug Well Water Consumers N = 30	Borehole Water Consumers N = 30	*p*-Value
Cd (µg/dL)	3.61 ± 0.55	2.66 ± 0.21	*p* = 0.001
Pb (µg/dL)	4.00 ± 0.26	2.08 ± 0.42	*p* = 0.001
Cu (µg/dL)	91.08 ± 1.41	106.70 ± 4.41	*p* = 0.001
Zn (µg/dL)	97.25 ± 2.16	116.95 ± 4.58	*p* = 0.001

Values are expressed in mean ± SD; Cd-cadmium, Pb-lead, Cu-Copper, Zn-Zinc. *p* < 0.05—Significant.

**Table 5:** Comparison of serum sex Hormone levels among Hand-dug Well/Borehole Water Consumers and Treated water consumers.

Measured Parameters	Hand-Dug Well/Borehole Water Consumers N = 60	Treated Water Consumers N = 30	*p*-Value
FSH (miu/mL)	3.26 ± 0.33	5.83 ± 0.38	*p* = 0.001
LH (miu/mL)	1.46 ± 0.14	6.98 ± 0.28	*p* = 0.001
E_2_ (pg/mL)	2.63 ± 0.33	9.93 ± 2.26	*p* = 0.002
PROG (ng/mL)	1.34 ± 0.41	4.11 ± 1.29	*p* = 0.04
T (ng/mL)	3.68 ± 0.30	6.54 ± 0.27	*p* = 0.001
PROL (ng/mL)	22.07± 0.66	18.37 ± 0.49	*p* = 0.001

Values are expressed in mean ± SD; FSH—follicle stimulating hormone, LH—Luteinizing hormone, E_2_—Estradiol, Prog—Progesterone, T—Testosterone, Prol—Prolactin. *p* < 0.05—Significant.

**Table 6:** Serum Hormone Levels among Hand-Dug Well and Borehole Water Consumers.

Measured Parameters	Hand-Dug Well Water Consumers N = 30	Borehole Water Consumers N = 30	*p*-Value
FSH (miu/mL)	1.92 ± 0.23	4.72 ± 0.74	*p* = 0.001
LH (miu/mL)	1.10 ± 0.11	2.21 ± 0.30	*p* = 0.001
E_2_ (pg/mL)	1.32 ± 0.27	4.38 ± 0.64	*p* = 0.001
PROG (ng/mL)	0.91 ± 0.27	1.93 ± 0.10	*p* = 0.244
T (ng/mL)	2.79 ± 0.16	4.98 ± 0.36	*p* = 0.001
PROL (ng/mL)	19.80± 0.76	25.85 ± 0.67	*p* = 0.001

Value are expressed in mean ± SD. FSH = follicle stimulating hormone, LH = Luteinizing hormone, E_2_ = Estradiol, Prog = Progesterone, T = Testosterone, Prol = Prolactin. *p* < 0.05—Significant.

**Table 7:** Interrelationship of sex hormones and other measured indices.

Variables	N	Regression Coefficient (β)	Standard Error of Coefficient (β)	Pearson Correlation Coefficient (r)	*p*-Value
FSH (miu/mL)					
Cd (µg/L)	60	0.067	0.096	−0.275	0.606
Pb (µg/L)	60	−0.260	0.019	−0.398	0.045 **
Cu (µg/dL)	60	−0.018	0.022	0.055	0.863
Zn (µg/dL)	60	0.304	0.005	0.422	0.0021 **
Age (years)	60	0.009	0.360	−0.015	0.930
BMI (kg/m^2^)	60	−0.062	0.064	−0.172	0.550
LH (miu/mL)					
Cd (µg/L)	60	−0.266	0.072	−0.622	0.005 **
Pb (µg/L)	60	−0.038	0.014	−0.493	0.674
Cu (µg/dL)	60	−0.055	0.016	0.033	0.452
Zn (µg/dL)	60	0.573	0.004	0.745	0.001 **
Age (years)	60	−0.034	0.027	−0.068	0.630
BMI (kg/m^2^)	60	0.022	0.648	−0.205	0.765

FSH = Follicle stimulating hormone, LH = Luteinizing hormone, E_2_ = Estradiol, Cd = Cadmium, Pb = Lead, Cu = Copper, Zn = Zinc, BMI = Body mass index, PROG = Progesterone, PROL = Prolactin, TESTO = Testosterone ** Significant (Pearson Correlation).

**Table 8:** Interrelationship of sex hormones with other measured indices.

Variables	N	Regression Coefficient (β)	Standard Error of Coefficient (β)	Pearson Correlation Coefficient (r)	*p*-Value
E_2_ (pg/L)					
Cd (µg/L)	60	0.066	0.287	−0.230	0.622
Pb (µg/L)	60	−0.026	0.057	−0.250	0.840
Cu (µg/dL)	60	0.040	0.065	0.065	0.703
Zn (µg/dL)	60	−0.399	0.016	0.412	0.003 **
Age (years)	60	−0.041	0.107	−0.041	0.687
BMI (kg/m^2^)	60	−0.131	0.191	−0.268	0.223
PROG (ng/mL)					
Cd (µg/L)	60	−0.170	0.183	−0.222	0.229
Pb (µg/L)	60	0.147	0.036	−0.055	0.287
Cu (µg/dL)	60	−0.075	0.041	−0.087	0.499
Zn (µg/dL)	60	−0.192	0.010	0.229	0.172
Age(years)	60	−0.022	0.068	0.004	0.841
BMI (kg/m^2^)	60	−0.036	0.081	−0.102	0.661

FSH = Follicle stimulating hormone, LH = Luteinizing hormone, E_2_ = Estradiol, Cd = Cadmium, Pb = Lead, Cu = Copper, Zn = Zinc, BMI = Body mass index, PROG = Progesterone, PROL = Prolactin, TESTO = Testosterone ** Significant (Pearson Correlation).

**Table 9:** Interrelationship of sex hormones with other measured parameters.

Variables	N	Regression Coefficient (β)	Standard Error of Coefficient (β)	Pearson Correlation Coefficient (r)	*p*-Value
PROL (ng/mL)					
Cd (µg/L)	60	−0.053	0.160	0.282	0.679
Pb (µg/L)	60	0.354	0.032	0.443	0.006 **
Cu (µg/dL)	60	0.093	0.036	−0.024	0.356
Zn (µg/dL)	60	−0.257	0.009	−0.404	0.047 **
Age (years)	60	0.061	0.060	0.098	0.654
BMI (kg/m^2^)	60	−0.046	0.106	0.072	0.654
TESTO (ng/mL)					
Cd (µg/L)	60	−0.132	0.087	−0.426	0.280
Pb (µg/L)	60	−0.105	0.017	−0.400	0.382
Cu (µg/dL)	60	−0.005	0.020	0.072	0.961
Zn (µg/dL)	60	0.418	0.005	0.542	0.001 **
Age (years)	60	0.006	0.033	−0.035	0.949
BMI (kg/m^2^)	60	0.054	0.058	−0.109	0.580

FSH = Follicle stimulating hormone, LH = Luteinizing hormone, E_2_ = Estradiol, Cd = Cadmium, Pb = Lead, Cu = Copper, Zn = Zinc, BMI = Body mass index, PROG = Progesterone, PROL = Prolactin, TESTO = Testosterone ** Significant (Pearson Correlation).

## Data Availability

Data from MSc project conducted at the Department of Medical Laboratory Science, University of Benin, Benin City, Nigeria.

## Abbreviations

Pb	lead
Cd	Cadmium
Zn	Zinc
Cu	Copper
FSH	Follicle Stimulating Hormone
LH	Luteinizing hormone
PROL	Prolactin
PROG	Progesterone
E_2_	Estradiol
T	Testosterone
GnRH	Gonadotropin-Releasing hormone
HPG	Hypothalamic-Pituitary-Gonadal
